# Fabrication of AlCoCrFeNi High-Entropy Alloy Coating on an AISI 304 Substrate via a CoFe_2_Ni Intermediate Layer

**DOI:** 10.3390/e21010002

**Published:** 2018-12-20

**Authors:** Wenyuan Cui, Sreekar Karnati, Xinchang Zhang, Elizabeth Burns, Frank Liou

**Affiliations:** 1 Department of Mechanical and Aerospace Engineering, Missouri University of Science and Technology, Rolla, MO 65409, USA; 2 Department of Metallurgical Engineering, Missouri University of Science and Technology, Rolla, MO 65409, USA

**Keywords:** high-entropy alloy, laser metal deposition, elemental powder, graded material

## Abstract

Through laser metal deposition, attempts were made to coat AlCoCrFeNi, a high-entropy alloy (HEA), on an AISI 304 stainless steel substrate to integrate their properties. However, the direct coating of the AlCoCrFeNi HEA on the AISI 304 substrate was found to be unviable due to cracks at the interface between these two materials. The difference in compositional change was suspected to be the source of the cracks. Therefore, a new transition route was performed by coating an intermediate layer of CoFe_2_Ni on the AISI 304 substrate. Investigations into the microstructure, phase composition, elemental composition and Vickers hardness were carried out in this study. Consistent metallurgical bonding was observed along both of the interfaces. It was found that the AlCoCrFeNi alloy solidified into a dendritic microstructure. The X-ray diffraction pattern revealed a transition of the crystal structure of the AISI 304 substrate to the AlCoCrFeNi HEA. An intermediate step in hardness was observed between the AISI 304 substrate and the AlCoCrFeNi HEA. The AlCoCrFeNi alloy fabricated was found to have an average hardness of 418 HV, while the CoFe_2_Ni intermediate layer had an average hardness of 275 HV.

## 1. Introduction

As a novel metallic alloy system, high-entropy alloys (HEAs) have received considerable attention in the past decade. The name HEA indicates that the mixing of the principal elements in the alloy leads to a substantial change in entropy. This change in entropy promotes the formation of a simple solid solution instead of complex compounds. One of the extensively studied HEAs is equiatomic AlCoCrFeNi, which shows high hardness, good wear behavior but low tensile ductility [1,2,3,4,5,6,7]. As-cast AlCoCrFeNi alloy showed a tensile elongation of 1.0%, while post-heat treatment, the elongation was increased to 11.7% [4]. Wang et al. studied the compressive properties of AlCrFeCoNi HEA prepared by vacuum arc melting. They found that this alloy showed large strain hardening and compressive strength up to 2004 MPa with a 32.7% compressive plasticity [6]. Munitz et al. reported the impact of heat treatment of AlCoCrFeNi HEA, in which the BCC (Body-centered cubic) matrix transformation occurred between 650 and 975 °C. This transformation led to a substantial increase in microhardness [5]. Further modification of this alloy system through the addition of titanium, leading to AlCoCrFeNiTi*_x_* (*x* = molar ratios), was found to be promising for wear protection [1]. Further, AlCoCrFeNi HEA solidified with dendritic and interdendritic microstructures due to elemental segregation. Dendritic segregation regions were found to be Al- and Ni-rich, while interdendritic areas were Fe- and Cr-rich, and the distribution of Co was uniform. Body-centered cubic (BCC) Fe and Cr precipitates, and B2 (ordered BCC) Al- and Ni-rich matrices were observed in previous studies [2,4,6,8,9]. Most of these studies are based on material fabricated through processes such as casting and arc melting. Unlike these early studies, laser metal deposition (LMD) was implemented in this study. 

LMD is capable of fabricating freeform three-dimensional metallic components [10,11,12] and has been used to fabricate several HEAs [12,13,14,15,16]. Chen et al. fabricated Al_x_CoFeNiCu_1-*x*_ (*x* = 0.25, 0.5 and 0.75 atom %, respectively) HEAs using elemental powders on the AISI 304 substrate. They reported an increase in hardness with an increase in aluminum content [16]. He et al. used laser cladding to produce FeCoCrNiAlTi*_x_* (*x* = 0, 0.25, 0.5, 0.75 and 1 atom %, respectively) coating on Q253 steel through the use of elemental powders. Addition of titanium was observed to improve the hardness and wear resistance of the HEA [15]. Similarly, FeCoCrAlCu HEA coating by laser cladding demonstrated good wear resistance under a dry sliding condition [17]. 

In this paper, the feasibility of coating an AlCoCrFeNi HEA on an AISI 304 stainless steel substrate was investigated. Sole LMD fabrication of AlCoCrFeNi HEA components is very costly due to the need for high-purity (i.e., 99.9%) raw powders of elements such as Co, Cr and Ni. AISI 304 stainless steel, on the other hand, is a low-cost structural material. However, AISI 304 is a soft material with low wear resistance. It is widely used in industrial facilities, transportation equipment and architectural applications. Therefore, by coating AlCoCrFeNi HEA on AISI 304, it can enhance the hardness of AISI 304 structures. This combination of materials could facilitate fabrication of components for applications that require both hardness and wear resistance. 

However, direct coating of AlCoCrFeNi HEA on AISI 304 is difficult due to the change in chemistry, thermal expansion and residual stress of the dissimilar materials. For example, the measured coefficient of thermal expansion (CTE, 10^−6^/K) for AlCoCrFeNi HEA was 9.03 (293–303 K), 12.47 (368–378 K) and 13.54 (423–773 K) [18]. However, the CTE values of AISI 304 were 14.7 (293 K), 16.3 (400 K), 19.5 (700 K) and 20.2 (800 K) [19]. Harihar et al. observed crack formation at the bottom of an AlCoCrFeNi deposit when deposited on an AISI 304 substrate. Due to the brittleness of the deposited material, the deposit broke off from the AISI 304 substrate easily [12]. An extensive network of cracks occurred when a TiVCrAlSi HEA was cladded on a Ti-6Al-4V substrate. This was attributed to the difference between the thermal expansion coefficients and residual stresses associated with the high cooling rate in laser cladding [20]. 

Therefore, to facilitate the dissimilar material bond, an intermediate layer was necessary and could accommodate the residual stresses and variation in chemistry change [10,21,22]. Intermediate layers of Fe/Cr/V were used between AISI 316 stainless steeland Ti-6Al-4V to facilitate a similar material bond [10]. Currently, there are few studies available identifying the viable intermediate layer between AlCoCrFeNi HEA and AISI 304. In this study, an attempt was made to coat the equiatomic AlCoCrFeNi HEA on the AISI 304 substrate using LMD. The objective was to obtain a strong bond between the two materials. We first demonstrated the issues with direct-coating the HEA onto the substrate. Then we proposed a candidate intermediate material and proved its viability. 

## 2. Materials and Methods 

Elemental powders of gas-atomized aluminum (Al), chromium (Cr), cobalt (Co), nickel (Ni) and iron (Fe) from Atlantic Equipment Engineers Inc. were used as precursor materials. These powders, weighed in required ratios, were mixed using a Turbula mixer (Glen Mills Inc., Clifton, NJ, USA) for 1 h to obtain homogeneous blends. Commercially procured AISI 304 bar stock (dimensions: 2.75 inch × 2 inch × 0.25 inch) was used as the substrate material for the deposition. The particle size distribution of the elemental powders stated by the producer is as tabulated in Table 1. Elemental analysis of the elemental powders is listed in Table 2. Elemental compositions (atom %) of the as-blended CoFe_2_Ni intermediate layer and AlCoCrFeNi alloy are given in Table 3. 

**Figure 1:**
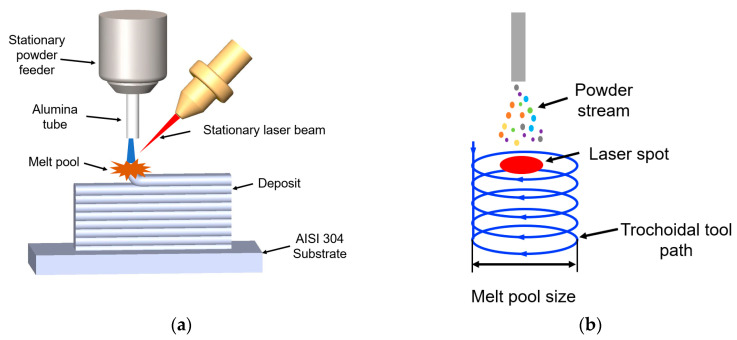
Schematic of the experimental setup, (**a**) laser metal deposition (LMD) system and (**b**) the trochoidal tool path.

The laser deposition process was performed in an LMD system whose schematic representation is as seen in Figure 1a. The heat source was a 1 kW continuous-wave YAG fiber laser (IPG Photonics, Oxford, MA, USA) with a 2 mm beam diameter. The powders were fed using a vibration X2 powder feed system procured from Powder Motion Labs. The powder was introduced into the melt pool through an alumina tube. A computer numerical control (CNC) table was used to facilitate the movement during the deposition. Argon gas was used to ensure an inert atmosphere and act as a carrier gas to deliver the powder mixture to the melt pool. 

In the current setup, the 2 mm spot size is insufficient to attain a large capture efficiency of the powder. This is due to the scatter of the powder flow out of the powder feed tube. This scatter was suspected to vary with individual precursor powder. Therefore, in order to obtain as-deposited compositions that are close to as-blended compositions, the capture efficiency during the deposition process needed to be increased. A trochoidal toolpath (shown in Figure 1b) was designed to create a large enough melt pool to improve capture efficiency during deposition. This toolpath was inspired by “weave”-style toolpaths that are commonly used in welding. 

The AISI 304 substrates were cleaned with acetone to remove the impurities such as dirt and oil from the surface. A preheating scan was conducted by running the laser across the substrate surface. To ensure a successful start, the power of the initial five layers of the deposition was carried out at 750 W and 8.5% (3.36 g/min) powder feed rate. The remainder of the deposit was run at a power level of 550 W and 8.5% (3.36 g/min) powder feed rate. The thickness of each layer is 1 mm. 

After laser deposition, vertical transverse sections of the specimens were cut using a wire electric discharge machine (Hansvedt Industries Inc., Rantoul, IL, USA) and mounted in Bakelite for polishing and etching. The metallographic specimens were first ground using 240, 400, 600 and 800 grit silicon carbide papers and then polished using 15 μm, 9 μm and 3 μm diamond suspensions. The final step of polishing involved 0.05 μm colloidal silica suspension. To reveal the microstructure, the electrolytic etching was carried out in the nitric acid solution (70 mL nitric acid, 30 mL distilled water) at 5 V for 5 seconds. Scanning electron microscopy (SEM), energy dispersive X-ray spectroscopy (EDS) and electron backscatter diffraction (EBSD) were performed on Helios Nanolab 600 SEM (Thermo Fisher Scientific, Waltham, MA, USA). The SEM image was acquired by an Everhart-Thornley detector. The EDS element was analyzed by the factory standardizations provided in the Aztec software. The EBSD step size was selected to be 2.5 μm. EBSD data acquisition and analysis were conducted using Aztec and Channel 5 software, respectively. Grain size was measured by the line intercept method, and the misorientation angle was 10°. Optical microscopy images were collected using a Hirox optical microscope. X-ray diffraction patterns were collected using Philips X’pert MRD using Cu anode. The Vickers hardness was measured using a Struers Duramin hardness tester (Struers Inc., Cleveland, OH, USA) at a 9.8 N load and a 10 s load duration. The reported hardness results were the average of three indentations. 

## 3. Results and Discussions 

### 3.1. Direct Coating of AlCoCrFeNi HEA on AISI 304 Substrate

**Figure 2:**
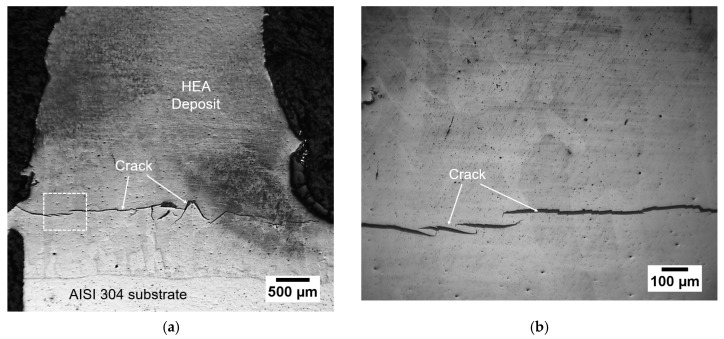
(**a**) Optical microscopy image of the vertical transverse cross-section of direct AlCoCrFeNi HEA coating on AISI 304 substrate, (**b**) a high-magnification view of the dashed–line-boxed area in (**a**).

The direct LMD of the AlCoCrFeNi HEA on the AISI 304 substrate will be discussed first. Figure 2a shows a portion of the vertical transverse section of the HEA deposit near the AISI 304 substrate. An area close to the crack zone, as marked in the dashed-line box, is shown in Figure 2b with high magnification. A network of cracks, mostly transverse and horizontal in orientation, were found to be prevalent. Cracking occurred at the bottom of this HEA deposit. This could be attributed to the mismatch between the thermal expansion coefficients. The CTE of this HEA was reported to be 9.03 (10^−6^/K, 293–303 K) while the value of AISI 304 was 14.7 (10^−6^/K, 293 K) [18,19]. 

**Figure 3:**
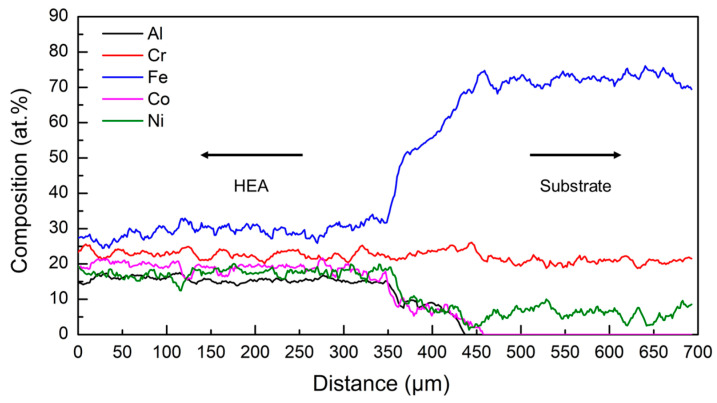
Elemental composition distribution along the interface between the AISI 304 substrate and the HEA deposit.

The elemental composition distribution along the interface between the HEA deposit and the AISI 304 substrate is shown in Figure 3. At the bottom of the melted metal, the composition mixing was significant during the laser deposition process (see Figure 3). The bottom of the deposit had high susceptibility of cracking in the transverse cross-section, as seen in Figure 2. 

**Figure 4:**
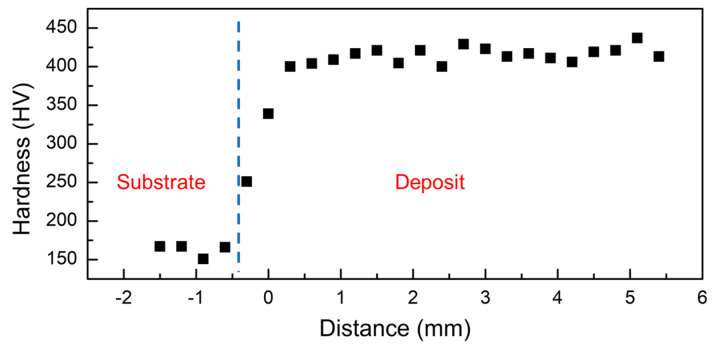
Vickers hardness profile of the direct coating of the AlCoCrFeNi alloy on AISI 304.

The variation in Vickers hardness across the HEA–AISI 304 direct coating is presented in Figure 4. The average Vickers hardness of the HEA deposits was 412 HV, while that of the substrate was 161 HV. Since the coefficients of thermal expansion are mismatched between HEA and the substrate, residual stresses were developed during the laser deposition process. The AISI 304 substrate had a high elongation rate from 28% to 50% in the temperature range of 300–500 °C [23]. However, the tensile elongations of the AlCoCrFeNi HEA were 1% (as-cast condition) and 11.7% (after heat treatment) [4]. A difference in ductility exists between the substrate and the HEA. Having an intermediate material to bridge these differences was deemed necessary. 

### 3.2. A New Transition Route

**Figure 5:**
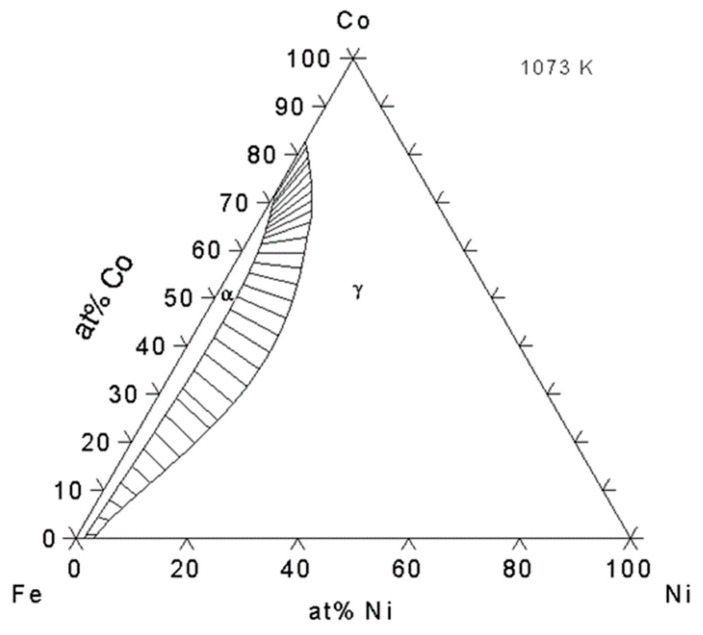
Ternary alloy phase diagram of Fe–Co–Ni at 1073 K [24].

A blend of Fe, Co and Ni powders was selected as the candidate intermediate material. Since they are among the constituents of the AlCoCrFeNi HEA, no special procurement was needed. A Fe–Co–Ni ternary phase diagram at 1073 K compiled from experimental data is shown in Figure 5 [24]. Fe, Ni and Co have excellent mutual solubility, and no brittle intermetallic phases are expected. From the phase diagram, an atomic composition ratio of Fe, Ni and Co of 50%, 25% and 25%, respectively, was chosen. The selected ratio is expected to bridge the material composition gap between the AlCoCrFeNi HEA and AISI 304. This new transition route, AISI 304 substrate → CoFe_2_Ni intermediate layer → AlCoCrFeNi HEA, was then carried out and characterized.

### 3.3. AlCoCrFeNi HEA–AISI 304 with an Intermdeiate Layer

#### 3.3.1. Microstructure

**Figure 6:**
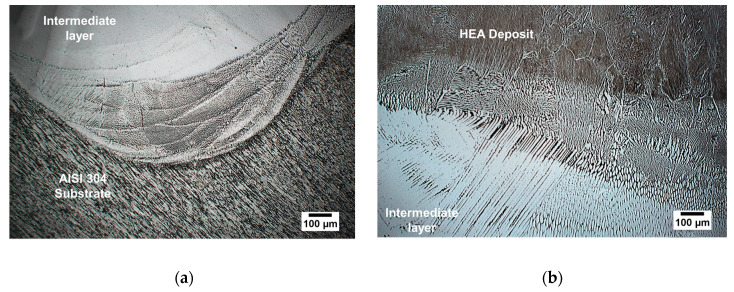
The optical microstructure of (**a**) the CoFe_2_Ni intermediate layer and the AISI 304 substrate and (**b**) the AlCoCrFeNi alloy deposit and the CoFe_2_Ni intermediate layer.

The CoFe_2_Ni intermediate layer was coated on the AISI 304 substrate using premixed elemental powder. Then, the AlCoCrFeNi HEA was coated on the intermediate layer by LMD. The intermediate layer composition was theorized to avoid the formation of intermetallic compounds and bridge the large gap in strength differences. Figure 6a,b shows the optical images of etched surfaces of transverse sections of these deposits. Unlike the HEA–AISI 304 direct coating, no apparent cracks were observed, which indicated an improvement in bonding. However, issues of microporosity persisted. A dendrite microstructure was observed along the interface between the intermediate layer and the HEA. 

**Figure 7:**
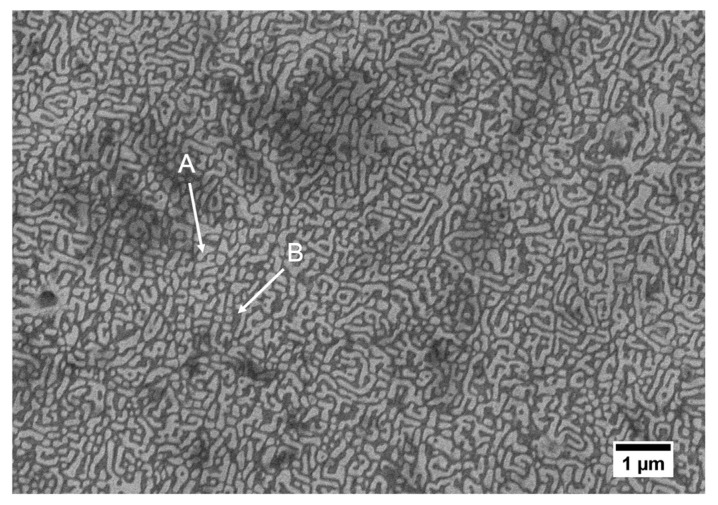
Secondary electron image of the AlCoCrFeNi HEA microstructure at a magnification of 10000.

A high-magnification secondary electron image of the AlCoCrFeNi HEA deposit is shown in Figure 7, where a two-phase dendritic microstructure was observed. The area fraction of the dendritic microstructure was ~52%, while the interdendritic area fraction was ~48%. The interdendritic region is named A, and the dendritic region is named B. The mean elemental compositions of A and B (average from three arbitrary points) were analyzed by EDS, and the results are listed in Table 4. It is shown that the atomic percentages of Al and Ni were ~29% in A and ~41% in B. The percentages of Fe and Cr were ~54 atom % in A and 43 atom % in B. These results indicate that Fe and Cr were rich in A, while Al and Ni were rich in B. The composition of Co did not show evident differences between A and B. The mixing enthalpies between Fe–Cr, Fe–Ni, Fe–Co, Fe–Al, Cr–Ni, Cr–Co, Cr–Al, Ni–Co, Ni–Al and Co–Al were −1, −2, −1, −11, −7, −4, −10, 0, −22 and −19 kJ/mol, respectively [6,25]. The mixing enthalpy of Al and Ni was higher than other pairs, which indicated that Al and Ni tended to form atomic pairs and segregate. Similar results have been reported for the AlCoCrFeNi HEA, with this microstructure being attributed to the spinodal decomposition [2,4,5,6,9]. 

**Figure 8:**
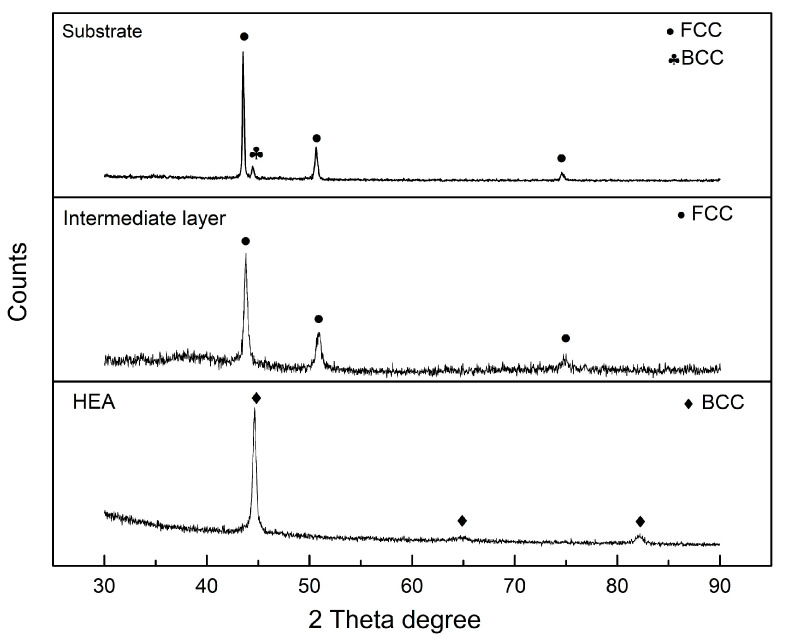
XRD pattern of the AISI 304 substrate, the CoFe_2_Ni intermediate layer and the AlCoCrFeNi HEA.

XRD was used to identify the crystal structures of the intermediate layers and the HEA. A transition of the crystal structure was observed from the AISI 304 substrate to the AlCoCrFeNi alloy. The XRD patterns of the AISI 304 substrate, the CoFe_2_Ni intermediate layer and the AlCoCrFeNi alloy are shown in Figure 8. The present phases and the corresponding crystallographic information are summarized in Table 5. The peak patterns of FCC were observed in the CoFe_2_Ni intermediate layer, while BCC peak patterns were detected in the AlCoCrFeNi alloy. Löbel et al. found BCC and B2 (ordered BCC) phases in AlCoCrFeNiTi*_x_* (*x* = 0) when fabricated via arc melting [1]. A similar result was reported by Shiratori et al., when casting was employed to produce an AlCoCrFeNi HEA [26]. Due to the same basic lattice structure and lattice parameters, the B2 ordered structure is very hard to detect from XRD, as the peak patterns of B2 and BCC are the same [2,9]. However, the evidence of the existence of the B2 phase was found from the EDS analysis above. Previously, an AlCoCrFeNi HEA was reported to also contain the FCC crystal structure with preheating or post-heat treatment [5,13,26]. The FCC structure was not found in this work, which could be because the high cooling rate during LMD inhibited the formation of the FCC crystal structure [5,13,26]. 

**Figure 9:**
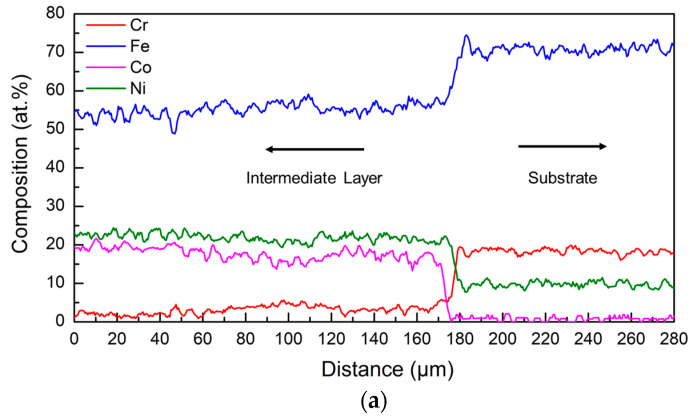

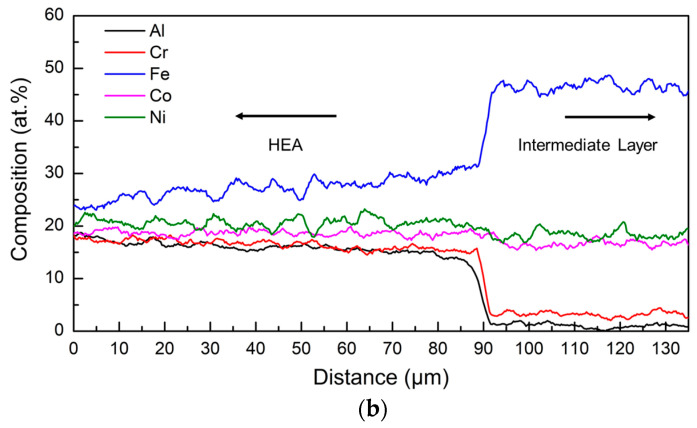
Elemental composition distribution along the boundary, (**a**) CoFe_2_Ni intermediate layer and AISI 304 substrate and (**b**) AlCoCrFeNi HEA and CoFe_2_Ni intermediate layer.

The evolution in chemistry from the intermediate layer to the substrate was characterized by an EDS line scan first. The quantitative results are shown in Figure 9a. The EDS measured results of the AISI 304 substrate (Cr: ~18–19 atom %, Fe: ~70–72 atom %, Ni: ~9–10 atom % in Figure 9a) did not vary from the nominal AISI 304 elemental compositions. Mn (~1–2 atom %) was detected in the AISI 304 substrate by EDS but is not shown in Figure 9. The percentages of Co (~17–22 atom %) and Ni (~21–23 atom %) reduced, while the Fe (~54–56 atom %) content increased from the intermediate layer to the AISI 304 substrate. A small amount of Cr (~3–5 atom %) was present in the intermediate layer, because the substrate was mixed with the intermediate layer. The composition distribution from the HEA to the intermediate layer is shown in Figure 9b. The constituents of the AlCoCrFeNi HEA were detected by EDS (Al: ~16–17 atom %, Co: 19–20 atom %, Cr: ~17 atom %, Fe: ~25 atom %, Ni: ~20–21 atom %). The difference between the as-blended (20 atom %) and as-deposited aluminum (~16–17 atom %) percentages is suspected to be a consequence of inconsistency in capture efficiencies of the constituent powders, and evaporation due to differences in melting point. Al and Cr were present in the intermediate layer as seen in Figure 9b, and their total content was ~4–5 atom %.

#### 3.3.2. EBSD

**Figure 10:**
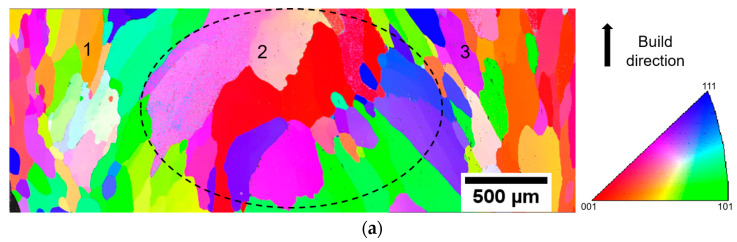

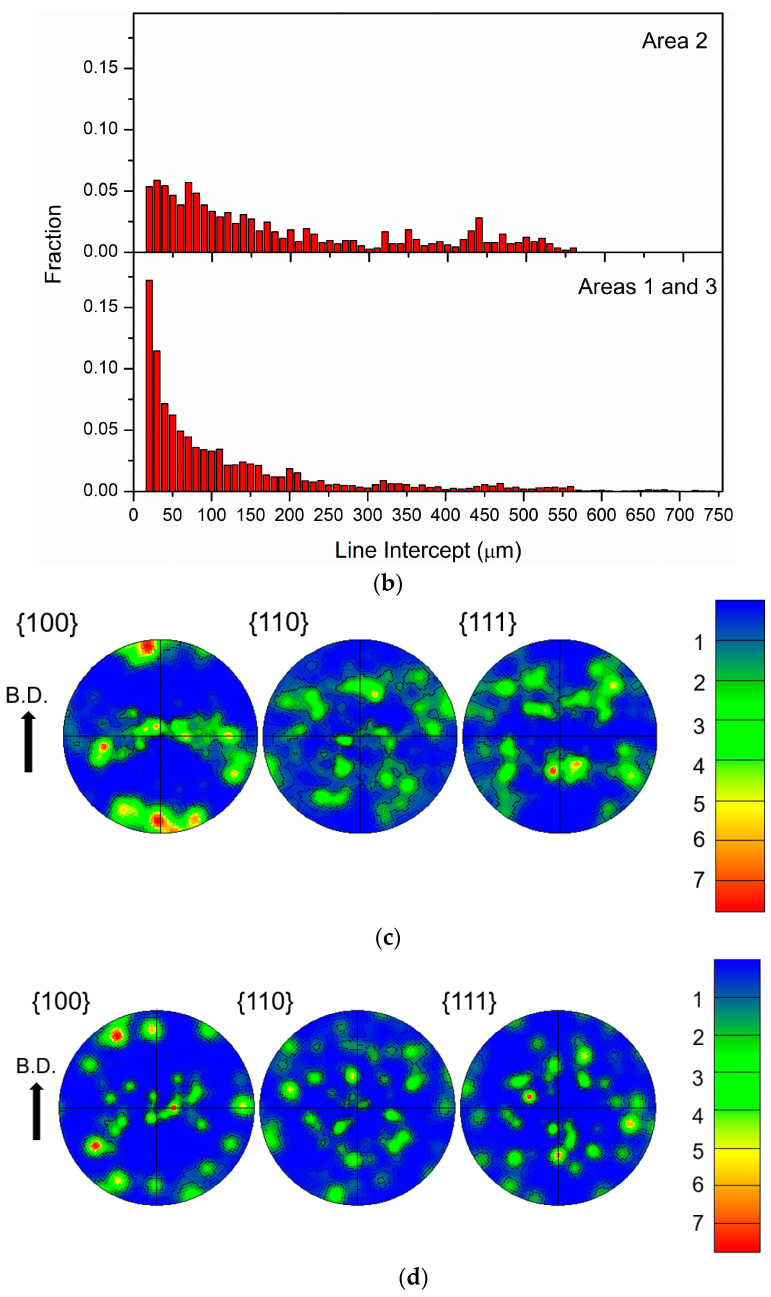
(**a**) Inverse pole figure (IPF)IPF map of the bottom of the HEA section in the specimen; the measured region was approximately 3.4 mm × 1.2 mm, from the left to the right side in the cross-section parallel to the build direction (BD); (**b**) distribution of the intercept length of grains with the bin size of 10 µm; (**c**) pole figure of areas 1 and 3; and (**d**) pole figure of area 2 in (**a**).

Figure 10a shows the inverse pole figure (IPF) map obtained from the bottom of the HEA section of the specimen. The measured area was approximately 3.4 mm × 1.2 mm of the cross-section parallel to the build direction (BD), which spanned from the left to the right of the specimen. The difference in color indicates the different crystallographic orientations. From Figure 10a, the overall constitution can be classified into two zones—the edge zone (1 and 3) and the middle zone (2). In areas 1 and 3, the grains were observed to be elongated along the build direction (see 1 and 3 in Figure 10a). The distributions of the intercept lengths (using 100 horizontal lines) in different areas are depicted Figure 10b. The median linear intercept for areas 1 and 3 was 72.5 µm, while it was 127.5 µm for area 2. From the linear intercept distribution of area 2, 25% of the intercept values were greater than 300 µm, whereas only 14% of the intercept values were above 300 µm for areas 1 and 3. This grain morphology is likely to be a consequence of deposition toolpath and variation in cooling rate at edges and in the middle [27,28]. Figure 10c,d show the {100}, {110} and {111} pole figures of different areas, which give the distribution of the pole density along the build direction. The pole figure of the areas 1 and 3 (Figure 10c) suggests that the orientations of the grains were close to the <100> direction. However, the grains were random in orientation and did not appear with obvious texture in area 2 (Figure 10d). Further study is necessary to investigate the impact of this toolpath on the grain morphology. 

#### 3.3.3. Vickers Hardness Analysis

**Figure 11:**
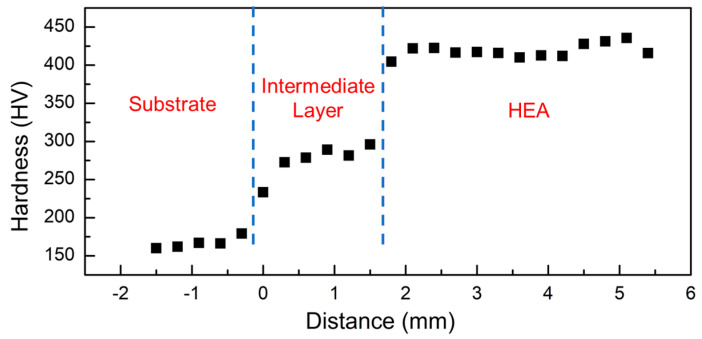
Vickers hardness profile of the AISI 304 substrate—AlCoCrFeNi HEA with the CoFe_2_Ni intermediate layer.

Figure 11 gives the Vickers hardness distribution of the AlCoCrFeNi HEA deposited on the AISI 304 substrate with the CoFe_2_Ni intermediate layer. The Vickers hardness of the CoFe_2_Ni intermediate layer was around the 275 HV, which could be attributed to the solid solution strengthening. Table 6 lists the Vickers hardnesses of the AlCoCrFeNi HEA, annealed AISI 304, aged Inconel 625, and annealed duplex steel SAF 2205 [29,30,31]. The average Vickers hardness of the HEA deposit was in the range of 418 HV, because of the second-phase strengthening [4]. 

According to the XRD results, the AISI 304 substrate and the CoFe_2_Ni intermediate layer had an FCC structure, while the AlCoCrFeNi HEA had a BCC structure. The transition from FCC to BCC structure is also expected to enhance the hardness. The high hardness is expected to correlate with good performance in strength and wear resistance [1,16].

## 4. Conclusions

An AlCoCrFeNi HEA was coated on an AISI 304 substrate by laser metal deposition (LMD) technology. The coating on the substrate without and with the intermediate layer was characterized and discussed. The main conclusions are as follows:

- Cracking was found to be prominent when the AlCoCrFeNi HEA was directly coated on the AISI 304 substrate due to the compositional change between HEA and the substrate.
- Using an intermediate layer of CoFe_2_Ni improved the bond. The incorporation of the intermediate layer successfully eliminated crack formation in the deposit.
- XRD patterns revealed a transition of crystal structure from FCC in the AISI 304 substrate to BCC in the AlCoCrFeNi alloy. The evidence of a B2 phase in the AlCoCrFeNi HEA was also found in the EDS analysis results.
- The AlCoCrFeNi alloy fabricated by LMD was found to have an average hardness of 418 HV, while the CoFe_2_Ni intermediate layer had an average hardness of 275 HV.

## Figures and Tables

**Table 1:** Particle size distribution of the precursor elemental powders.

Materials	US Standard Mesh
Al	−100
Co	−100/+325
Cr	−100
Fe	−100
Ni	−100/+325

**Table 2:** Elemental analysis (atom %) of elemental powders as provided by the manufacturer.

Materials	Al	Cr	Si	Fe	C	Ni	Co	S	Ca
Al	0.88	-	0.07	0.05	-	-	-	-	-
Co	-	-	-	0.002	-	<0.001	~0.99	-	0.001
Cr	-	0.89	-	0.02	0.09	-	-	-	-
Fe	-	-	-	0.99	0.01	-	-	-	-
Ni	-	-	-	0.01	0.05	0.92	-	0.02	-

**Table 3:** Nominal compositions (atom %) of CoFe_2_Ni and AlCoCrFeNi alloy powder blends.

Alloy	Al	Co	Cr	Fe	Ni
CoFe_2_Ni	0	25	0	50	25
AlCoCrFeNi	20	20	20	20	20

**Table 4:** Elemental compositions analyzed by energy dispersive X-ray spectroscopy (EDS)of the AlCoCrFeNi HEA shown in Figure 7.

Elements (atom %)	Al	Co	Cr	Fe	Ni
A	16.2	16.8	23.4	30.2	13.4
B	23.5	15.7	19.4	24.2	17.2

**Table 5:** Summary of phases detected by XRD analysis for AISI 304, CoFe_2_Ni and the AlCoCrFeNi HEA.

Alloy	Lattice	Structure	Space Group	Lattice Parameter (Å)
AISI 304	FCC	Cu	Fm-3m (225)	3.5911
	BCC	Fe	Im-3m (229)	2.87
CoFe_2_Ni	FCC	Cu	Fm-3m (225)	3.5911
AlCoCrFeNi HEA	BCC	W	Im-3m (229)	2.876

**Table 6:** Vickers hardness of various alloys.

Alloy	Hardness (HV)	Reference
AlCoCrFeNi HEA	418	This work
AISI 304 annealed	188	[29]
Inconel 625 aged	225	[30]
Duplex steel SAF 2205 annealed	290	[31]
